# Long-Lasting Antidepressant Action of Ketamine, but Not Glycogen Synthase Kinase-3 Inhibitor SB216763, in the Chronic Mild Stress Model of Mice

**DOI:** 10.1371/journal.pone.0056053

**Published:** 2013-02-04

**Authors:** Xian-Cang Ma, Yong-Hui Dang, Min Jia, Rui Ma, Fen Wang, Jin Wu, Cheng-Ge Gao, Kenji Hashimoto

**Affiliations:** 1 Department of Psychiatry, First Affiliated Hospital of Medical College of Xi'an Jiaotong University, Xian, China; 2 Department of Forensic Medicine, Key Laboratory of the Health Ministry for Forensic Medicine, Key Laboratory of Environment and Genes Related to Diseases of the Education Ministry, Xi'an Jiaotong University School of Medicine, Xi'an, China; 3 Division of Clinical Neuroscience, Chiba University Center for Forensic Mental Health, Chiba, Japan; University of Regensburg, Germany

## Abstract

**Background:**

Clinical studies demonstrate that the *N*-methyl-D-aspartate (NMDA) receptor antagonist, ketamine, induces rapid antidepressant effects in patients with refractive major depressive disorder and bipolar depression. This rapid onset of action makes ketamine a highly attractive drug for patients, particularly those who do not typically respond to therapy. A recent study suggested that glycogen synthase kinase (GSK)-3 may underlie the rapid antidepressant action of ketamine, although the precise mechanisms are unclear. In this study, we examined the effects of ketamine and GSK-3 inhibitor SB216763 in the unpredictable, chronic mild stress (CMS) mouse model of mice.

**Methodology/Principal Findings:**

Adult C57/B6 male mice were divided into 2 groups, a non-stressed control group and the unpredictable CMS (35 days) group. Then, either vehicle, ketamine (10 mg/kg), or the established GSK-3 inhibitor, SB216763 (10 mg/kg), were administered into mice in the CMS group, while vehicle was administered to controls. In the open field test, there was no difference between the four groups (control+vehicle, CMS+vehicle, CMS+ketamine, CMS+SB216763). In the sucrose intake test, a 1% sucrose intake drop, seen in CMS mice, was significantly attenuated after a single dose of ketamine, but not SB216763. In the tail suspension test (TST) and forced swimming test (FST), the increased immobility time seen in CMS mice was significantly attenuated by a single dose of ketamine, but not SB216763. Interestingly, the ketamine-induced increase in the sucrose intake test persisted for 8 days after a single dose of ketamine. Furthermore, a single administration of ketamine, but not SB216763, significantly attenuated the immobility time of the TST and FST in the control (non-stressed) mice.

**Conclusions/Significance:**

These findings suggest that a single administration of ketamine, but not GSK-3 inhibitor SB216763, produces a long-lasting antidepressant action in CMS model mice.

## Introduction

Accumulating evidence suggests that dysfunction of the glutamate neurotransmitter system is associated with the pathophysiology of mood disorders, such as major depressive disorder (MDD) and bipolar disorder (BP) [1]–[7]. Levels of glutamate are altered in blood, cerebrospinal fluid, and brains of patients with MDD and BP [8]–[15]. Furthermore, clinical studies including double-blind, placebo-controlled randomized trials, demonstrate the efficacy of a single dose of the ionotrophic *N*-methyl-D-aspartate (NMDA) receptor antagonist, ketamine, in treating patients with refractory MDD and bipolar depression [16]–[19]. The rapid onset of ketamine action makes it a highly attractive drug for patients with mood disorders, some of whom do not typically respond to therapy, although, the mechanistic action is unknown [20]–[24].

Several lines of evidence suggest a role for glycogen synthase kinase-3 (GSK-3) in the pathophysiology of mood disorders, such as MDD and BP [25]–[28]. This serine/threonine kinase phosphorylates glycogen synthase, inhibiting glycogen synthesis. Studies show that GSK-3 inhibitors, including L803-mts, AR-R014418 and NP031115 produce antidepressant effects in rodents [29]–[31]. Interestingly, Beurel et al. reported that a subanesthetic dose (10 mg/kg) of ketamine reduced symptoms of depression in mice subjected to the depression model of learned helplessness [32]. They also found that ketamine increased the serine-phosphorylation of GSK-3α and GSK-3β, two isoforms of GSK-3. Furthermore, the antidepressant effect of ketamine was absent in GSK-3α^21A/21A^/β^9A/9A^ knock-in mice; these mice contain serine-to-alanine mutations which eliminate inhibitory serines within GSK-3α/β [32]. These results appeared to suggest that GSK-3 inhibition may mediate the antidepressant action of ketamine [32]. However, the antidepressant effect of ketamine was detected 24 hours after administration, whereas phosphorylation occurred 30 and 60 minutes after dosing [32].

The purpose of this study was, therefore, to investigate the effects of ketamine and GSK-3 inhibitor in the mouse model of unpredictable chronic mild stress (CMS). This model is widely used as a preclinical animal model of depression [33], [34]. Specifically, we examined whether ketamine or the established GSK-3 inhibitor, SB216763 [35], [36], could improve depressive-like behavior seen in mice subjected to a model of unpredictable CMS [37]. In addition, we also examined the antidepressant effects of ketamine and SB216763 in control (non-stressed) mice.

## Materials and Methods

### Animals

Sixty six adult male C57 BL/6J mice (aged: 7±1 weeks; average body weight: 20±2 g) were purchased from Vital River Laboratories (Beijing, PR China). Mice were housed singly in cages (26 cm×18 cm×13 cm) under a controlled 12-hour/12-hour light-dark cycle (lights on: 7:00 a.m.), with a room temperature of 21±2°C and humidity of 55±5%. Mice were given free access to water and food. The experimental protocols (Permit Number: 200910011) were approved by the Xi'an Jiaotong University Laboratory Animal Administration Committee, and performed according to the Xi'an Jiaotong University Guidelines for Animal Experimentation. Experiments also conformed to the Guide for the Care and Use of Laboratory Animals published by the US National Institutes of Health. All efforts were made to minimize suffering.

**Figure 1:**
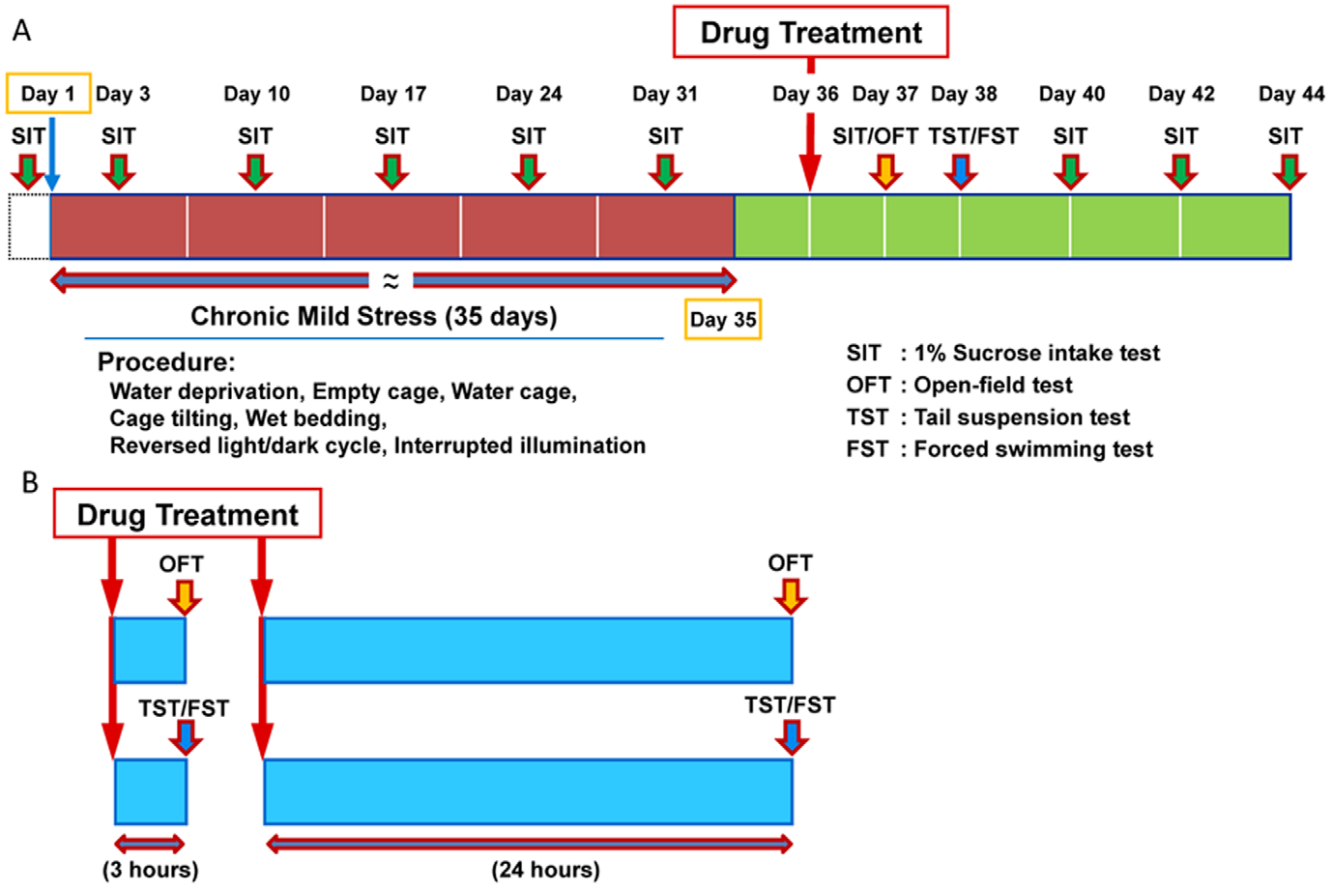
Experimental protocol. (**A**): CMS paradigm. CMS procedures were performed for 5-weeks. Drug treatment was performed day 36. The 1% sucrose intake test (SIT) was performed at baseline, days 3, 10, 17, 24, 31, 37, 40, 42 and 44. Vehicle, ketamine (10 mg/kg), or SB216763 (10 mg/kg) were administered at day 36. The open field test (OFT) was performed at day 37. The tail suspension test (TST) and the forced swimming test (FST) was performed at day 38. (**B**): Acute effects of ketamine and SB216763 in control mice. Vehicle, ketamine (10 mg/kg, i.p.), or SB216763 (2.5, 5.0, or 10 mg/kg, i.p.) were administered into control mice. The behavioral tests, OFT, TST, and FST, were performed 3 and 24 hours after a single administration.

The unpredictable CMS model was implemented according to our previous method with a slight modification [37] (**Figure 1**). Mice were allowed to adapt to stable environmental conditions for 1 week, and then a baseline of 1% sucrose solution consumption was measured for 2 weeks, 3 times per week (on Mondays, Wednesdays and Fridays), during the hours of 9:00–10:00 a.m. When a stable baseline of sucrose consumption was achieved, mice were divided into 4 groups: controls plus vehicle, CMS mice plus vehicle, CMS mice plus ketamine, and CMS mice plus SB216763. Thirty four mice were assigned to the groups of non-stressed control mice (n = 8) or mice subjected to CMS (35 days) procedures (n = 26). There were no significant baseline differences in sucrose consumption and body weight amongst the animals. One day after CMS training (day 36), vehicle (10 ml/kg; 10% DMSO), ketamine (10 mg/kg, Fujian Gutian Pharmaceutical CO. LTD., China), or SB216763 (10 mg/kg, Tocris Bioscience, UK) was administered intraperitoneally (i.p.) into mice. The dose (10 mg/kg) of ketamine was used since this dose was effective in the animal models of depression [32], [37], [38]. The dose (10 mg/kg) of SB216763 was used in the CMS paradigm since the dose (5–7.5 mg/kg) was effective in the behavioral abnormalities in male CD-1 mice after administration of amphetamine and cocaine [40], [41]. Doses of 2.5, 5.0 and 10 mg/kg of SB216763 were used in control mice.

### CMS paradigm

Mice in the CMS model received a variety of stressors, including 45°cage tilting, cage-switching, empty cage, soiled cage, continuous overnight illumination, inversion of the light/dark cycle and lights on/off for a short period of time, as reported previously [37] (**Figure 1**). These stressors were applied semi-randomly, during both light and dark periods. Mice in the CMS group were paired randomly with each other and housed together for 1 hour, once a week. These mice showed aggressive behavior (e.g., biting attack, lateral threat, aggression and tail rattle) when two previously isolated mice were placed in the same cage. The CMS paradigm procedures spanned a 5 week period.

### Behavioral tests

Behavioral tests were performed in the following order: open-field test and 1% sucrose intake test on day 37 and tail suspension test (TST) and forced swimming test (FST) on day 38. The 1% sucrose intake test was also performed on days 40, 42, and 44. Mice were put into the test room 30 minutes before testing. All tests were performed between 9:00 a.m–17:00 p.m. in a quiet room. After each test, mice were replaced in their individual cages and returned to the breeding room.

### Open-field test

The open-field test was performed 24 hours (on day 37) after a single dose of vehicle, ketamine (10 mg/kg), or SB216763 (10 mg/kg). In accordance with previous methods [37], the apparatus consisted of a square box with dimensions, 45 cm×45 cm×45 cm. Mice were placed into the center of the open box under a dark light (25 lx), and allowed to explore the arena for 1 hour, between the hours of 11:00 a.m–16:00 p.m. A video-computerized tracking system (SMART, Panlab SL, Barcelona, Spain) was used to record the distance traveled, as a measure of locomotor activity.

### Tail suspension test (TST)

TST was performed 48 hours (on day 38) after a single dose of vehicle, ketamine (10 mg/kg), or SB216763 (10 mg/kg), as reported previously [37]. Mice tails were wrapped with tape to cover approximately 4/5 of the tail length and fixed upside down on a hook. The immobility time of each mouse was recorded over a 6 minute period. Since 7 mice in the TST climbed up their tails during the 6-min test session, data were not obtained from these seven mice, as reported previously [42].

### Forced swimming test (FST)

FST was performed 48 hours (on day 38) after a single dose of vehicle, ketamine (10 mg/kg), or SB216763 (10 mg/kg), as reported previously [37]. Equipment for this test consisted of a glass barrel (high×diameter: 25 cm×15 cm) with 10 cm of water, at a temperature of approximately 22±1°C. A mouse was placed in this barrel and immobility time was measured for 6 minutes, using a video surveillance system. After testing, mice were removed into a normal heat preservation breeding cage with padding, and covered with an absorbent towel. The cage was then placed in an electric dryer, at 30–35°C, for approximately 20 minutes.

### Sucrose intake test

The sucrose intake test was performed 24 hours (on day 37), 4 days (day 40), 6 days (day 42), and 8 days (day 44) after a single dose of vehicle, ketamine (10 mg/kg), or SB216763 (10 mg/kg), as reported previously [37]. After the CMS procedure was started, 1% sucrose intake was measured between 9:00–10:00 a.m., every Wednesday. Fourteen hours before the sucrose intake test, all mice (including the control group) were deprived of water and all CMS procedures were halted. Mice resumed drinking freely after the sucrose intake test. Control mice were kept under the same laboratory conditions, in different chambers.

### Behavioral effects of ketamine and SB216763 in control mice

We examined the antidepressant effects of ketamine and SB216763 in control mice. Vehicle (10 ml/kg), ketamine (10 mg/kg) or SB216763 (2.5, 5.0, or 10 mg/kg) were administered i.p. into control mice. Behavioral evaluations (locomotion, TST, FST) were performed at 3 or 24 hours after a single administration (**Figure 1**).

### Statistical analysis

The data are expressed as the mean±standard error of the mean (S.E.M.), and data analyses were performed using PASW Statistics 18 (formerly SPSS statistics; SPSS, Tokyo, Japan). Data for the sucrose intake test were analyzed by repeated measures analyses of variance (ANOVA), and one-way ANOVA, followed by *post hoc* Fisher's PLSD test. Behavioral data, including open field test, TST, and FST, were analyzed by one-way ANOVA, followed by *post hoc* Fisher's PLSD test. *P* values of less than 0.05 were considered statistically significant.

## Results

**Figure 2:**
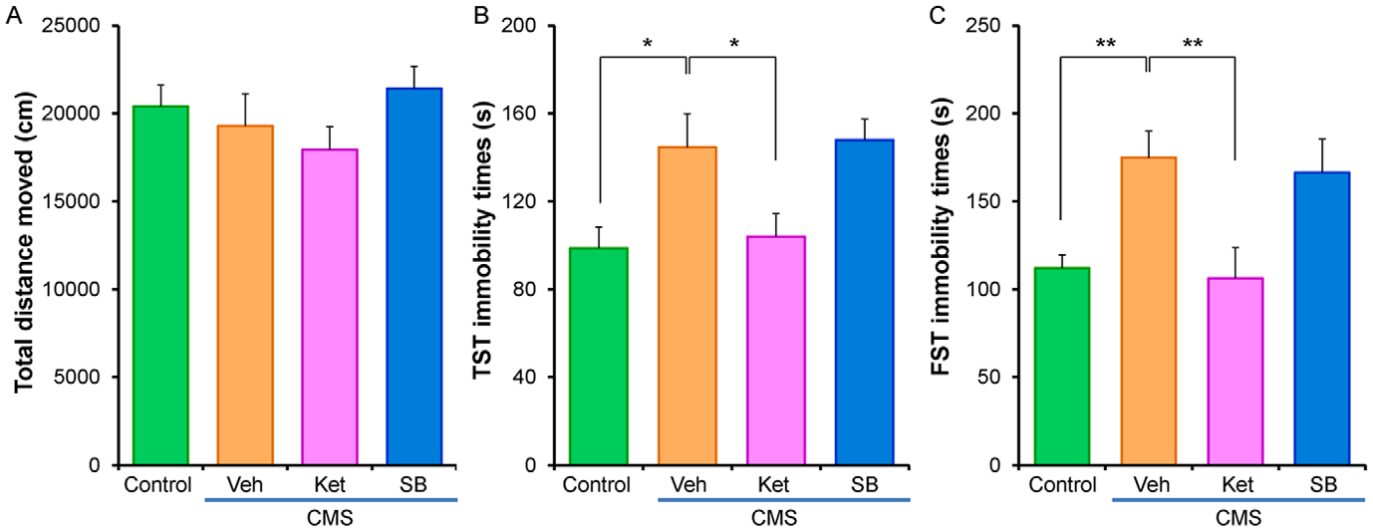
Effects of ketamine and the established GSK-3 inhibitor SB216763 in the CMS model. (**A**) Locomotion: There were no differences between the four groups. Data show the mean±SEM (n = 8 or 9). (**B**) Tail-suspension test (TST): The increased immobility time of mice in the CMS groups, decreased significantly 48 hours (day 38) after a single dose of ketamine (10 mg/kg, i.p.), but not SB216763 (10 mg/kg, i.p.). Data show the mean±SEM (n = 5–8). (**C**) Forced swimming test (FST): The increased immobility time of mice in the CMS groups decreased significantly 48 hours (day 38) after a single dose of ketamine (10 mg/kg, i.p.), but not SB216763 (10 mg/kg, i.p.). Data show the mean±SEM (n = 8 or 9). *p<0.05, **p<0.01 as compared to CMS+Vehicle group.

Figure 1 shows the experimental protocol of CMS (35 days) model of mice. We examined the effects of ketamine (10 mg/kg) and SB216763 (10 mg/kg) on depression-like behavior in the CMS model. In the open field test, there was no difference (F [3], [30] = 0.748, p = 0.532) between the four groups (**Figure 2A**). In the tail-suspension test (TST), one-way ANOVA analysis revealed that immobility times were significantly different (F [3], [23] = 4.685, p = 0.011) between the four groups. *Post hoc* testing showed that ketamine (10 mg/kg), but not SB216763 (10 mg/kg), significantly (p = 0.018) attenuated total immobility time for the TST in CMS mice (**Figure 2B**). In the forced-swimming test (FST), one-way ANOVA analysis revealed that immobility times were significantly different (F [3], [30] = 5.473, p = 0.004) between the four groups. *Post hoc* testing showed that ketamine (10 mg/kg), but not SB216763 (10 mg/kg), significantly (p = 0.003) attenuated total immobility time in CMS model mice undergoing the FST (**Figure 2C**).

**Figure 3:**
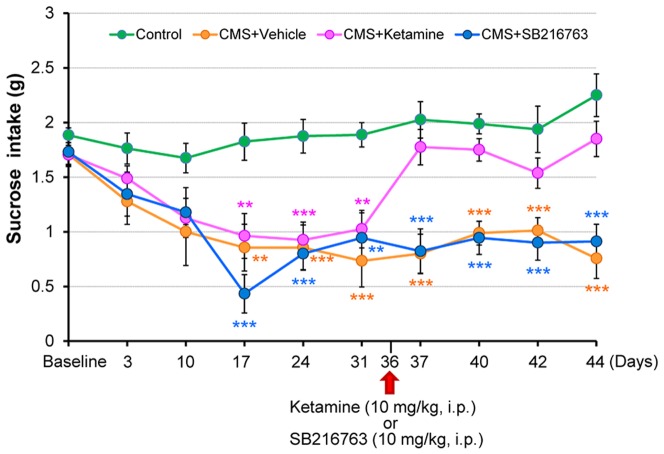
Effects of ketamine and the established GSK-3 inhibitor SB216763 in the anhedonia model. The decreased intake of 1% sucrose in the CMS groups was significantly attenuated 24 hours, 4 days, 6 days and 8 days after a single dose of ketamine (10 mg/kg, i.p.), but not of SB216763 (10 mg/kg, i.p.). Data show the mean±SEM (n = 8 or 9). **p<0.01, ***p<0.001 as compared to Control group.

In rodents, the unpredictable CMS paradigm produced anhedonia-the loss of interest in normally pleasurable and rewarding activities, which is a core symptom of depression [37], [43]–[45]. Repeated ANOVA analysis revealed that the intake of 1% sucrose solution was significantly different (F [9, 270] = 6.409, p<0.001) in the four groups (**Figure 3**). Subsequent one-way ANOVA and *pot hoc* testing showed that a reduction of 1% sucrose intake by mice in the CMS model was significantly improved by a single dose of ketamine (10 mg/kg), but not SB216763 (10 mg/kg). Interestingly, this improvement was still detectable 8 days after a single administration of ketamine (**Figure 3**).

**Figure 4:**
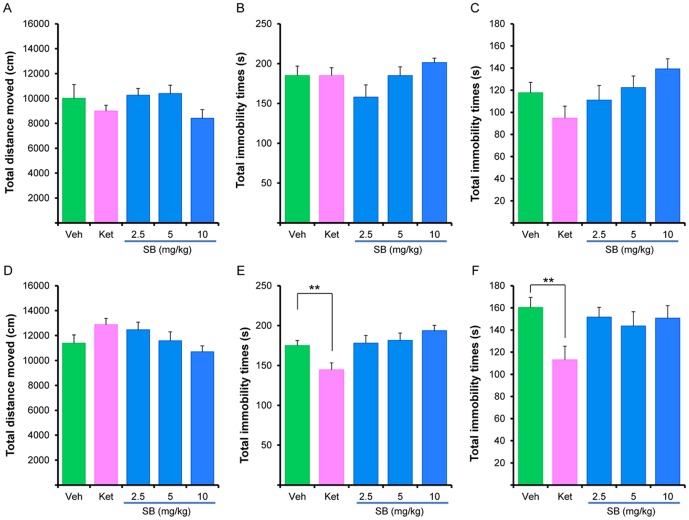
Effects of ketamine and SB216763 on control mice. Behavioral tests in control mice were performed 3 hours and 24 hours after a single administration of vehicle, ketamine (10 mg/kg, i.p.) or SB216763 (2.5, 5.0, or 10 mg/kg, i.p.). (A): Locomotion: There were no differences between the five groups. Data show the mean±SEM (n = 14–16). (B) Tail-suspension test (TST): There were no differences between the five groups. Data show the mean±SEM (n = 13–16). (C) Forced swimming test (FST): There were no differences between the five groups. Data show the mean±SEM (n = 13–15). (D) Locomotion: There were no differences between the five groups. Data show the mean±SEM (n = 15 or 16). (E) Tail-suspension test (TST): Ketamine significantly (p = 0.001) decreased immobility time, 24 hours after administration. Data show the mean±SEM (n = 15 or 16). (C) Forced swimming test (FST): Ketamine significantly (p = 0.037) decreased immobility time, 24 hours after administration. Data show the mean±SEM (n = 15 or 16). *p<0.05, **p<0.01 as compared with the control group.

We examined the antidepressant effects of ketamine and SB216763 in control (non-stressed) mice. First, we performed behavioral evaluations, 3 hours after a single administration of ketamine (10 mg/kg) or SB216763 (2.5, 5.0, or 10 mg/kg). In the open field test, one-way ANOVA analysis revealed no differences (F [4, 65] = 1.208, p = 0.315) between the five groups (**Figure 4A**). In the TST, one-way ANOVA analysis revealed was no differences (F [4, 61] = 2.231, p = 0.308) between the five groups (**Figure 4B**). Similarly in the FST, one-way ANOVA analysis revealed no differences (F [4, 65] = 1.886, p = 0.124) between the five groups (**Figure 4C**).

Next, we performed behavioral evaluations 24 hours after a single dose of ketamine (10 mg/kg) or SB216763 (2.5, 5.0, or 10 mg/kg). In the open field test, one-way ANOVA analysis revealed no differences (F [4, 73] = 2.184, p = 0.079) between the five groups (**Figure 4D**). In contrast, in the TST and FST, one-way ANOVA analysis highlighted significant differences (TST: F [4, 69] = 5.114, p = 0.001, FST: F [4, 73] = 2.703, p = 0.037) between the five groups (**Figures 4E and 4F**). Subsequent analysis showed that ketamine (10 mg/kg), but not SB216763, significantly (TST: p = 0.001, FST: p = 0.037) decreased immobility time in control mice, 24 hours after administration (**Figure 4E and 4F**).

## Discussion

In this study, we found that ketamine (10 mg/kg), but not SB216763 (10 mg/kg), exerted antidepressant-like effects, as scored in the TST, FST, and anhedonia tests, in mice subjected to the CMS model. This effect was detectable at 24 hours, and interestingly, in CMS-induced anhedonia, it was still detectable 8 days after a single dose of ketamine. Li et al. [39] reported that in the sucrose preference test, ketamine (10 mg/kg, i.p.) ameliorated anhedonia in rats under the CMS model (21 days), and that it produced a long-lasting (up to 7 days) increase in sucrose preference, relative to untreated CMS rats. The rapid and long-term antidepressant effects of ketamine in this CMS model are similar in time course to the therapeutic effects seen patients with refractory MDD and bipolar depression [16]–[19].

SB216763, a potent and selective GSK-3 inhibitor is reported to cross the blood-brain barrier after i.p. administration [46]. Pretreatment (20 minutes before) with SB216763 (5 mg/kg), attenuated amphetamine-induced ambulation and stereotypy, and behavioral sensitization in mice [40]. Furthermore, SB216763 (2.5–7.5 mg/kg) attenuated cocaine-induced hyperactivity [41], but only partially attenuated hyperactivity produced by SKF-82958 [47]. These results suggest that systemic administration of SB216763 could inhibit GSK-3 in the brain. However, we could find no antidepressant effect for SB216763 in the mouse CMS model and control mice, although the dose used in this study could cause GSK-3 inhibition in the brain. A recent study showed that intracerebroventricular injection of SB216763 attenuated behavioral abnormalities (e.g., locomotion, rotarod performance, prepulse inhibition, novel object recognition, and duration of loss of righting reflex) in mice that had been administered ketamine [48], suggesting that SB216763 is capable of blocking the effects of ketamine in mice. Taken together, it is unlikely that a direct inhibition of GSK-3 is involved in the rapid antidepressant action of ketamine in the CMS mouse model, although a further study using lower doses is needed.

Previous reports showed that the selective, brain-permeable GSK-3 inhibitor, AR-A014418, decreased FST immobility time in control rats [30]. However, treatment with AR-A014418 resulted in a spontaneous and generalized reduction in locomotor activity in mice, indicating that this induced reduction in activity constitutes its therapeutic action [30]. The effects of AR-A014418 were detectable as early as 30 minutes after a single dosing, although behavioral tests were not performed until 24 hours after dosing [30]. In this study, we found no antidepressant effect for ketamine or SB216763 in control mice, at the 30 minute time point, after a single dosing, in contrast with previous reports [29]–[31]. The reasons for this discrepancy are currently unclear. However, we found that ketamine, but not SB21673, showed antidepressant activity in control mice 24 hours after a single administration, suggesting that ketamine induces long-lasting antidepressant effects in control mice.

In this study, no acute experiments using SB216763 in the CMS model were performed within the earlier time frame, after a single dosing. Therefore, it would be interesting to examine the earlier time effects of ketamine and SB216763 in the CMS model. It would also be intriguing to examine whether chronic administration of SB216763 exerts an antidepressant effect in the CMS model. As mentioned previously, the effects of ketamine were detectable from 24 hours to 8 days after a single dosing, even though ketamine would no longer be present in the body, due to rapid clearance [39]. Although this possibility was raised by Beurel et al. [32], we could find no evidence of an antidepressant-like effect for SB216763 in the CMS mouse model and control mice. It was reported that ketamine increases the phosphorylation of GSK-3, and that mice with a knock-in mutation that blocks this phosphorylation, do not respond to ketamine in a depression model [32]. In addition, it has been shown that the NMDA receptor antagonists, such as phencyclidine and dizocilpine, transiently could increase GSK-3β activity and increase the active (phosphor-tyrosine-216) forms of GSK-3β and decrease the inactive (phospho-serine-9) forms in the rat forebrain [49], [50]. Further detailed studies are therefore, required to determine the mechanisms underlying the induction of GSK-3 phosphorylation by ketamine.

In the two experiments (CMS model and control mice), the immobility time of TST and FST was different (ures 2 and 4). It is well known that behavioral results using TST and FST could be affected by a number of variability factors (e.g., season, circadian rhythm, environmental of the laboratory, gender, housing of animals, observer, strain, test/retest)[51]. In this study, these two experiments were performed in different laboratory rooms and different seasons, and were also performed by different observers. Therefore, these factors may contribute to this difference of the immobility time for two experiments.

In conclusion, these findings show that while ketamine produces a rapid and enduring antidepressant effect in the CMS mouse model, the GSK-3 inhibitor, SB216763, does not. It is therefore unlikely that within the CMS model, a direct inhibition of GSK-3 may underlie the antidepressant mechanisms of ketamine.
